# The impact of de-familization on green innovation: Evidence from SRDI family firms in China

**DOI:** 10.1371/journal.pone.0314110

**Published:** 2025-01-10

**Authors:** Jiarong Huang, Lixin Zhou, Shuai Song, Hanwei Zhou

**Affiliations:** 1 Institute for Chengdu-Chongqing Economic Zone Development, Chongqing Technology and Business University, Chongqing, China; 2 Chongqing Finance and Economic College, Chongqing, China; 3 Business School, Southwest University of Political Science & Law, Chongqing, China; Zhejiang University of Technology, CHINA

## Abstract

Green innovation is essential for sustainable development, especially in China’s Specialized-Refined-Differentiated-Innovative (SRDI) enterprises. Family-owned SRDI firms, in particular, have attracted attention due to their de-familization strategies and their influence on green innovation. Our study analyzes panel data from 2016 to 2021 for listed SRDI family firms to investigate how de-familization in management rights and ownership impacts green innovation. Using socio-emotional wealth (SEW) theory and a fixed-effects model, we find that de-familization significantly negatively affects green innovation, with corporate governance serving as a mediating factor. Digital transformation moderates these negative effects, while market concentration exacerbates them. These impacts are more pronounced in firms before being designated as "Little Giants," those receiving higher government subsidies, those located in eastern regions, or those not classified as major polluters. This research provides actionable insights for SRDI family firms to strategically manage de-familization, optimize resource allocation, implement customized governance strategies, and promote sustainable growth.

## 1. Introduction

Currently, nations worldwide face significant challenges like resource scarcity and environmental pollution in their pursuit of economic development [1]. Achieving a balance between economic growth and environmental preservation is a global goal for sustainable development, and green innovation is a crucial strategy to meet these objectives [2, 3]. "Invisible champion" enterprises serve as the backbone of technological breakthroughs and international market dominance in developed nations like Germany. Similarly, SRDI small and medium-sized enterprises (SMEs) are key tools for the Chinese government in addressing these challenges. SRDI is a multidimensional, multi-tiered concept that broadly refers to enterprises that are specialized, refined, distinctive, and innovative. Among them, the "Little Giant" SRDI enterprises are the most outstanding, and their development can fundamentally drive the optimization and upgrading of the industrial structure of China’s manufacturing sector. The Chinese government places great importance on cultivating SRDI SMEs, emphasizing the need to foster a group of "Little Giant" SRDI enterprises that focus on niche fields and possess both innovative capabilities and growth potential. Additionally, the government stresses the need to increase support for green technology innovation among SMEs, fostering SRDI SMEs and "Little Giant" enterprises in the green technology innovation domain. Therefore, an in-depth exploration of the factors influencing the green innovation behaviors of SRDI "Little Giant" enterprises can not only help formulate "excellence cultivation programs" for these enterprises but also serve as a model and guide for ordinary firms.

According to the lists of the first four batches of SRDI "Little Giant" enterprises released by the Ministry of Industry and Information Technology, there are 735 listed companies on the A-share market, 80% of which possess varying degrees of family attributes. Family attributes represent a unique inheritance and governance mechanism. Moreover, as small and medium-sized enterprises, SRDI firms are more likely to be influenced by these family characteristics. Over the past 40 years of reform and opening up, these firms have made invaluable contributions to the rapid development of the Chinese economy. Nonetheless, as the founders of these family firms gradually age, a significant number of firms established during the early stages of reform and opening up are currently undergoing transformation and reform challenges. Certain second-generation heirs of family firms are gradually assuming the family legacy, while in other instances, family firms are broadening their pool of management personnel from core family members to non-family members. Furthermore, with the progress of listed family firms, the controlling family equity undergoes continuous dilution. The phenomenon of de-familization is gradually escalating, and this process is protracted. Existing research suggests that fueled by the pursuit of SEW, family firms exhibit a greater propensity to partake in green innovation endeavors [4]. As family firms within SRDI SMEs progressively undertake the trajectory of de-familization, will their green innovation behavior be impacted? Answering this question helps explain how de-familization affects governance and also impacts the future of green innovation in SRDI family firms.

Presently, academic research on green innovation frequently begins by investigating influencing factors, including external factors such as environmental regulations [5, 6] and internal factors such as executive characteristics [7, 8]. Research on de-familization frequently concentrates on outcomes, such as corporate performance [9, 10], company value [11], innovation activities [12], risk-taking [13]. Despite its importance, the family aspect of SRDI firms has been overlooked. The effects of de-familization on green innovation are significant and should be further explored for theoretical insights into SRDI firms.

This study utilizes panel data from Chinese SRDI family firms covering the period from 2016 to 2021. By employing SEW theory and a two-way fixed-effects model, we empirically assess the impact of management de-familization (*DFM*) and ownership de-familization (*DFO*) on green innovation. Our research investigates the masking effect of corporate governance on the relationship between de-familization and green innovation, as well as the moderating effects of digital transformation and market concentration within this context. We aim to elucidate the mechanisms and contextual factors through which de-familization influences green innovation. Furthermore, we examine the heterogeneity of de-familization’s effects on green innovation based on several factors, including whether a company is included in the "Little Giant" list, the level of government subsidies received, the geographic region of the company, and its classification as a key polluter. **This paper contributes incrementally in multiple ways: *Firstly*,** it focuses on SRDI family firms, thereby broadening the scope of research on this subject. Existing literature has primarily concentrated on SRDI enterprises in general or conventional family firms, with limited investigation into family firms within the SRDI context. ***Secondly*,** it enhances and refines the examination of factors influencing green innovation. ***Thirdly*,** this study explores the mechanisms through which de-familization impacts green innovation via the pathway of "de-familization-corporate governance-green innovation," while also assessing how internal digital transformation strategies and external market concentration moderate this relationship. ***Finally*,** building on the policy framework surrounding SRDI, we analyze the heterogeneous impacts of de-familization on green innovation across different contexts. This analysis provides valuable insights for enterprises in strategizing their de-familization processes and implementing green innovation strategies. Additionally, it serves as a reference point for government efforts to enhance the institutional environment.

## 2. Literature review

### 2.1. De-familization

Family control stands as the most prominent characteristic distinguishing family firms from non-family firms. However, with the evolution of modern corporate systems and the development of family firms after going public, these enterprises require the integration of high-level professionals with specialized knowledge and management expertise, as well as the utilization of diversified capital. This entails an appropriate degree of de-familization to meet the demands of business development. The influence of the controlling family on the company undergoes a process from significant to diminishing and ultimately disappearing, which is considered a tangible manifestation of de-familization.

Some researchers utilize the proportion of non-family members in senior management as a metric to gauge the level of de-familization [14, 15]. Some scholars also investigate de-familization from the perspective of shareholding ratios [16, 17]. This paper primarily explores the discussion of de-familization in the context of management rights and ownership. De-familization of management rights (DFM) entails an augmentation in the proportion of non-family members within the management team [18–20]. De-familization of ownership (DFO) entails the gradual dilution of family equity, involving actions such as family members divesting or bringing in external investors [15, 21, 22]. Presently, a growing number of Chinese family firms are undertaking the process of de-familization.

### 2.2. Green innovation

Fussler defines green innovation as an innovative endeavor centered on mitigating environmental pollution and promoting social benefits. It encompasses innovation across multiple domains, including science and technology, production processes, and product development [23]. We assert that green innovation entails enterprises proactively integrating environmental awareness and responsibility into their daily production and operations. It encompasses a range of innovative activities across products, management, and other domains, aiming to achieve a harmonious integration of economic, social, and ecological benefits. It simultaneously considers two attributes: innovation and environmental protection [24]. The attribute of innovation is characterized by long-term and complex features, while the attribute of environmental protection demands low energy consumption, low emissions, recyclability, higher technological and financial thresholds, and longer payback periods. External institutional factors such as environmental regulations, market demand for green products, green finance [25], digital finance [26], as well as internal characteristics such as executives’ environmental awareness, executive overseas experience, bring about significant risks and uncertainties to green innovation.

### 2.3. The impact of de-familizationon green innovation

#### 2.3.1. De-familization and innovation

In recent years, there has been growing attention to innovation in family firms [27, 28]. Current research primarily compares family firms with non-family firms regarding factors influencing innovation behavior [17, 29], innovation inputs [30], etc. However, there has been limited exploration of the impact of de-familization on innovation. Duran et al. (2016) argue that family firms have an advantage in innovation output, while other scholars suggest that family involvement in management negatively impacts innovation [15], while non-family shareholder governance has a significantly promoting effect on corporate innovation [24].

#### 2.3.2. De-familization and environmental protection

Existing research consistently suggests that family members prioritize non-economic objectives such as corporate image and reputation [31], which align with a sustainable long-term orientation towards ecological environmental protection. Family-controlled listed companies are believed to exhibit better environmental performance compared to non-family firms, driven by the aim to safeguard their SEW [4]. On the other hand, non-family members, like professional managers and external investors, focus on the company’s short-term gains. They pay less attention to the family business’s pursuit of enduring success. Compared to family members, they show less concern for the environment and take fewer eco-friendly actions. They are also less likely to sacrifice short-term profits for the sake of green environmental objectives.

## 3. Research hypotheses

### 3.1. The impact of de-familizationon green innovation in SRDI family firms

SRDI enterprises are known for their technical expertise, precision, unique offerings, and continuous innovation. Adding family elements increases the likelihood of green innovation. Theories of long-term orientation and SEW strongly back this idea. Long-term orientation is widely acknowledged as a pivotal characteristic that distinguishes the goals of family firms from those of non-family firms [31]. Driven by the objective of ensuring the long-term growth of the enterprise, founding families prioritize not only economic interests but also the establishment and preservation of the company’s image, family reputation, social standing, intergenerational succession, and other non-economic benefits in daily management and strategic decision-making [4], sometimes at the expense of economic interests. SEW serves as the fundamental theory for analyzing the organization and behavior of family firms [32]. It denotes the non-economic value that businesses provide to fulfill familial emotional needs. Family members exhibit a strong inclination towards long-term SEW, emphasizing non-economic objectives such as family image and reputation. Consequently, they exhibit heightened attention to environmental concerns and undertake proactive measures to mitigate pollution.

However, as successive family executives withdraw and family ownership gradually dilutes, The original long-term orientation of family members [4] and stewardship behaviors such as generous dedication [31] gradually disappear. In its place emerges a fervent pursuit of the SRDI goals driven by the introduction of professional managers and the equity involvement of external investors, coupled with a preference for short-term financial performance. They adhere to the principles of SRDI in exercising power and fulfilling obligations, while maintaining the belief in "long-term sustainability" becomes increasingly challenging. They prioritize actions such as on-the-job consumption or short-term arbitrage to maximize their own interests, showing less concern for family emotions and blood relations.de-familization also indicates a shift away from the long-term orientation of family control, suggesting a diminished emphasis on non-economic benefits such as long-term corporate image and reputation by controlling families. They may exhibit a stronger inclination towards SRDI technological breakthroughs, thereby dominating markets in their specialized fields. Moreover, they might hesitate to engage in green innovation activities characterized by a long-term orientation, resulting in a reduction or slowdown in the level of green innovation. Interest misalignment and information gaps between non-family members and family members can further limit the company’s ability to innovate greenly.

#### 3.1.1. The impact of DFM on green innovation

It is increasingly common to observe key positions, such as senior management roles, in family businesses being filled by non-family professional managers [18]. The senior management personnel in SRDI SMEs possesses high levels of cognitive endowments, motivational cognition, and capability cognition [33]. Non-family executives among them often possess deeper industry experience, functional expertise, and management skills distinct from those of family members [18, 34]. However, due to their weaker emotional ties to the firm, non-family executives exhibit a diminished commitment to the long-term objectives of family heritage and corporate reputation, as suggested by the SEW theory [35].

Therefore, non-family executives are more likely to utilize their skills and knowledge to advance the company’s SRDI initiatives, while also enhancing corporate performance. Green innovation, from decision-making to value realization, often demands several years of sustained effort. This is particularly challenging for SRDI SMEs, typically small and resource-constrained, as it entails continuous R&D investment over an extended period [24], squeezing financial resources and potentially affecting profitability. Such circumstances may also tarnish the reputation of non-family executives in the executive market. Moreover, green innovation entails high uncertainty [36], posing the risk that non-family executives may invest significant economic resources during their tenure without achieving any tangible outcomes. Professional managers aim to mitigate operational and financial risks to protect their professional reputation [37]. Due to a focus on the opportunistic motivation of SRDI and the pursuit of immediate benefits, non-family managers may hinder the progress of green innovation in SRDI SMEs. Therefore, the following hypothesis is proposed:

**H1a:**DFM has a significantly negative impact on green innovation in SRDI family firms.

#### 3.1.2. The impact of DFO on green innovation

In the initial phases of family firms development, ownership of the enterprise usually rests predominantly with the controlling family [35]. As the institutional environment and market conditions evolve, and the enterprise expands to a certain extent, exclusive control of corporate ownership by family members becomes less conducive to its development. Consequently, controlling families frequently initiate equity reduction processes [35]. During this process, as the proportion of non-family shareholding gradually increases, their decision-making power regarding corporate strategy also increases. The founding family, aspiring to pass the enterprise down through generations, tends to prioritize the company’s long-term development. They may even prioritize upholding the company’s image and fulfilling social responsibilities over economic interests [38]. However, non-family shareholders, who typically prioritize their own interests [11], demonstrate significantly lower emotional attachment and sense of responsibility toward the enterprise compared to family members. Therefore, as non-family shareholders accumulate equity, the likelihood of them avoiding green innovation in their decision-making increases. On the other hand, continuous decreases in the family’s shareholding percentage due to DFO result in the family losing adequate control and influence over the enterprise. Consequently, the family faces challenges in prioritizing non-economic goals, such as emotional attachment and social responsibility, due to this loss of control [39]. In essence, while family members may aspire to uphold the family image and reputation through green innovation, the diminishing influence of the family over the enterprise complicates the realization of this goal. Moreover, excessive DFO, characterized by significant divestment by family shareholders, suggests a lack of confidence in the company’s future prospects. Such actions release a concerning signal that the controlling family plans to exit, resulting in heightened financial constraints for the enterprise. Under these circumstances, the implementation of green innovation, which demands significant investment and entails high risks, becomes even more challenging.

Ownership signifies critical control in family firms [35]. Consequently, when a company experiences DFO resulting from equity dilution and subsequent loss of influence, the gradual transfer of ownership control to non-family shareholders presents challenges for family members in allocating resources towards green innovation under the oversight of non-family shareholders. This consequently results in a decreased likelihood of initiating green innovation. Therefore, the following hypothesis is proposed:

**H1b:** DFO has a significantly negative impact on green innovation in SRDI family firms.

### 3.2. Mediating effect

Corporate governance encompasses internal and external mechanisms within a company [40]. De-familization improves corporate governance standards. DFM introduces professional managers with specialized knowledge and extensive governance experience into the enterprise. These managers establish an efficient management control system for the company, resulting in enhanced supervisory and incentive effects of corporate governance. The ownership structure constitutes one of the primary mechanisms of corporate governance [41]. DFO diversifies the ownership structure, and the inclusion of external investors can additionally strengthen the supervisory mechanisms in corporate governance [42].

Given its high input and high-risk nature, the implementation of a green innovation strategy largely relies on enterprises’ resources and willingness to green innovation. Effective corporate governance can establish monitoring and incentive mechanisms that are essential for promoting green innovation. The monitoring mechanism curbs managerial self-interest, mitigates management’s short-sightedness in neglecting environmental responsibility and green innovation, and fosters willingness for green innovation. External investors enhance supervisory oversight, mitigate conflicts of interest between majority and minority shareholders, and mitigate agency costs. Incentive mechanisms incentivize investors to actively engage in corporate investment decisions [43, 44], thereby bolstering corporate financial resources. SRDI enterprises have the resources and confidence to adopt green innovation strategies without losing focus on their main operations. Strengthening corporate governance can further promote green innovation within these companies.

Nevertheless, as de-familization advances, the proliferation of professional managers increases steadily. Their willingness for green innovation remains low due to self-interest and short-sighted behavior. Although some improvement may occur under the oversight mechanism, achieving a complete reversal is challenging. They still tend to focus more on the development of the SRDI core business. Simultaneously, external investors may not comprehend the company’s operational intricacies to the same extent as internal personnel. Consequently, they maintain a cautious stance towards investing in green innovation, thereby constraining financial resources allocated to green innovation, despite some alleviation. In summary, while corporate governance can partially mask the adverse effects of de-familization on green innovation, it cannot entirely rectify the short-sighted behavior of professional managers resulting from a deficiency in SEW, nor can it entirely alleviate resource constraints on green innovation. Therefore, de-familization continues to exert a detrimental influence on green innovation, albeit partially masked by the role of corporate governance, which acts as a mitigating factor. We put forth the following hypotheses:

**H2a:** Corporate governance plays a mediating role between de-familization and green innovation.
**H2b:** The mediating role of corporate governance between de-familization and green innovation manifests as a "masking effect".

### 3.3. Moderating effects

#### 3.3.1. The moderating effect of digital transformation

Digital transformation involves enterprises leveraging digital technology to enhance information processing efficiency, promote resource integration, and trigger transformations in production methods, organizational structure, and management processes. Through this, it systematically realizes the stimulation of innovation potential, cost savings in transactions, and the systematic acquisition of business value [45]. SRDI SMEs typically have smaller scales and relatively short establishment histories. Severe information asymmetry leads to higher agency costs and necessitates higher financing costs for SRDI SMEs [46]. Additionally, breakthroughs in SRDI technologies depend on substantial capital investment. Green innovation also demands significant financial support, and the positive impacts on energy conservation, emission reduction, and enhanced corporate image require considerable time to materialize. Confronted with financial pressures and funding challenges, non-family executives and shareholders of SRDI enterprises tend to shy away from the high investments, risks, and uncertainties linked with green innovation. Digital transformation can enhance performance and reduce costs to some extent, thereby mitigating financial challenges and subsequently diminishing non-family members’ resistance to green innovation.

Firstly, it improves financial performance [47]. Digital technology has greatly facilitated information dissemination [47], enhancing communication between upstream and downstream enterprises in the supply chain as well as within a company. It effectively integrates demand information from upstream and downstream enterprises with various data generated during the internal production and operational activities of the company [48]. It improves the allocation of enterprise resources, thereby enhancing financial performance. Simultaneously, it signals to the external environment that the company possesses positive prospects for development, significantly reducing the risk of stock price collapse. The increased confidence of non-family investors can attract more external investments to the company [49]. Secondly, it reduces investor transaction costs [50]. Digital transformation can enhance transparency in corporate information, reducing external transaction costs such as investor information search and contract signing. Thirdly, it decreases monitoring costs. Digital transformation facilitates family members in monitoring key indicators and the latest financial data. This visual management process helps compress opportunistic spaces for non-family executives, lowering the supervision costs of the enterprise. Fourthly, it lowers information communication costs. The ease of information dissemination brought about by digital technology significantly streamlines communication between enterprises [51]. In conclusion, digital transformation boosts the confidence of non-family managers and shareholders. They no longer need to allocate all limited resources to core technological research and development due to financial constraints. While there may still be a bias towards the core business, having sufficient funds allows for the allocation of some financial support to green innovation. Therefore, digital transformation alleviates the reluctance of non-family members towards green innovation. We propose the following hypothesis:

**H3a:** Digital transformation will weaken the negative impact of de-familization on green innovation.

#### 3.3.2. The moderating effect of market concentration

The centralization of market structure results in differences in market share and enterprise scale, thus presenting varying motivations and effects for green innovation [52]. With the increase in market concentration, there emerge specialized enterprises with higher market shares and larger scales [53]. These enterprises possess more substantial financial resources and are able to recruit a greater number of talented individuals, thereby establishing a robust core competitive advantage in their technical domain. Furthermore, economies of scale further propel the emergence of a situation where the stronger enterprises become even stronger [54]. In comparison, small enterprises are at a disadvantage in terms of resources and capabilities. They often lack strong independent research and original innovation capabilities in specialized fields, remaining in the mid to low-end of the industry chain. These small enterprises have inherently weaker resource foundations, and as market concentration increases, they face heightened survival pressure. Financing constraints become more significant, and their ability to withstand risks weakens. The immediate priority for these small enterprises is to innovate in product technology and compete for market share while maintaining their survival. However, engaging in challenging green innovation significantly increases costs, squeezes capital investment, and reduces profitability.

In this scenario, the increasing presence of non-family managers and shareholders intensifies the urgency for developing the core business. Since these non-family members lack the motivation to pursue SEW, the motivation for green innovation is further weakened. Thus, in situations of high market concentration, small enterprises undergoing de-familization encounter difficulties in managing resource conflicts between technical advancements and green innovation. Non-family members, unable to reconcile these conflicting pursuits aligned with SEW and long-term orientation [55], are disinclined to offer adequate and continuous backing for green innovation. Consequently, we propose the following hypothesis:

**H3b:** Market concentration strengthens the negative relationship between de-familization and green innovation.

**Fig 1:**
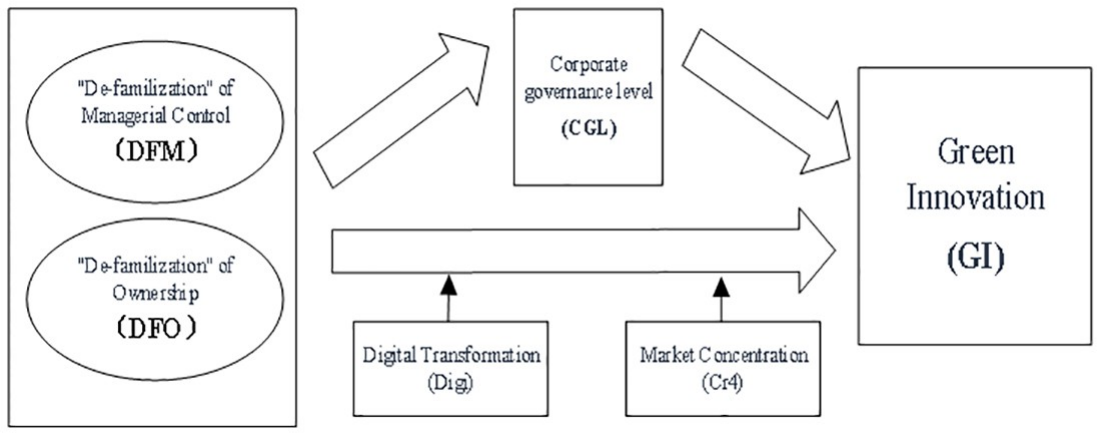
The logical framework diagram.

In summary, the research framework of this study is depicted in Fig 1.

## 4. Research design

### 4.1. Data source and sample selection

#### 4.1.1. Data source

The data for this study primarily originates from SRDI family firms listed on the A-share market. Among them, companies meeting the following criteria are classified as family firms: (1) Ultimate control can be attributed to an individual or a family unit. (2) The family (individual) directly or indirectly holds 20% or more of the shares of the listed company. Alternatively, if the family (individual) serves as the chairman or general manager, their shareholding exceeds 10%. If neither of the above two conditions is met, but the family (individual) holds the largest shareholding of the listed company, reaching the critical 10% threshold, and there is no second-largest shareholder with a shareholding exceeding the 10% threshold. (3) The ultimate controller is either directly or indirectly the largest shareholder of the listed company [22, 56].

Information on family members’ kinship relationships, shareholding situations, and executive team positions was obtained using the following methods: (1) Annual reports, prospectuses, and listing announcements were collected from the WIND Information website. (2) Information regarding the main controller, family shareholding, and family executives is disclosed in the "Shareholders and Actual Controllers" and "Basic Information of Directors, Supervisors, and Senior Management" sections. (3) Due to some companies not disclosing the kinship relationships among company shareholders and executives, we used search engines such as Baidu and Google to ascertain the relationships between individuals listed in the "Top 10 Shareholders" and "Basic Information of Directors, Supervisors, and Senior Management" sections of the annual reports and the actual controllers. When information could not be determined using these methods, additional data collection was conducted online to obtain more comprehensive information, and the final results were manually compiled. Additional data were sourced from the China National Research Data Service Platform (CNRDS) and the Guotai An Database (CSMAR).

#### 4.1.2. Sample selection

We conducted sample selection according to the following process: (1) Compile the list of national-level SRDI "Little Giants" for the years 2019–2022. (2) Exclude non-listed companies from the sample. (3) Exclude samples designated as ST and *ST. (4) Exclude samples that did not meet the criteria for family firms, while retaining samples that initially met the criteria but underwent changes later. (5) Considering the completeness of research variable data and the balance of panel data, we maintained the sample period from 2016 to 2021, resulting in a total of 786 enterprise-year observations, including 131 family firms. Data processing and analysis were performed using Stata 17 econometric analysis software.

### 4.2. Definition and measurement of variables

#### 4.2.1. Dependent variable

*Green innovation (GI)*. Existing studies commonly use green patents to characterize GI. Green invention patents, as opposed to green utility patents, more intuitively reflect a company’s capacity to produce green innovations. Additionally, it takes 3–5 years for a patent to be approved from the time of application. Considering the lag in patent approval, patent application data is more stable and timely compared to approved data. Therefore, we employ the natural logarithm of the sum of independent and collaborative green invention patent applications plus 1 to assess the level of green innovation in family firms for the current year [24, 57]. The green patent data is obtained from the Patent Search System of the National Intellectual Property Administration.

#### 4.2.2. Independent variables

*DFM*. Measured by the proportion of non-family executives, specifically the percentage of non-family members serving as general managers, deputy managers, and financial managers among the total number of executives. We initially obtain the total number of executives through annual reports. By combining information from the annual reports and manually collecting data from online sources, we identify the number of family members serving as senior management personnel. This includes blood relatives and in-laws such as parents, spouses, children, sons-in-law, daughters-in-law, etc. The difference between the total executive team size and the number of family members serving as senior management represents the current number of non-family executives.

*DFO*. Represented by the proportion of shares held by non-family individuals or institutions in the company. In this study, the percentage of shares held by family members in family enterprises was manually compiled through the analysis of annual reports of listed companies to derive the non-family ownership proportion.

#### 4.2.3. Mediating variable

*Corporate governance level (CGL)*. We adopt the methodology proposed by Bai et al. (2005) to measure CGL. Indicators selected encompass the perspectives of shareholders, the board of directors, and incentive mechanisms, including the shareholding ratio of the largest shareholder, ownership balance, property rights nature, institutional investor shareholding, board independence, board structure, management shareholding ratio, and the compensation of the top three management members to construct a comprehensive index. We employ the first principal component analysis method for measurement.

#### 4.2.4. Moderating variables

*Digital transformation (Digi)*. Drawing inspiration from the research report "Research Report on the Evaluation of Digital Transformation Index of Chinese Listed Companies" by the Wu Fei team, this paper derives the Digital Transformation Index from five dimensions: Artificial Intelligence Technology (A), Big Data Technology (B), Cloud Computing Technology (C), Blockchain Technology (D), and Digital Technology Application [49, 58, 59].

*Market concentration (Cr4)*. We measure industry concentration by calculating the sum of market shares held by the 4 largest companies in the industry.

#### 4.2.5. Control variables

Drawing on previous research findings in the exploration of family firms and green innovation, this study primarily selects various enterprise characteristics as control variables. Table 1 summarizes the main variables utilized in this study.

**Table 1:** Variables and description.

Classification	Variable	Symbols	Variable definition
Dependent variable	Green innovation	*GI*	See above for details
Independent variables	De-familization of management	*DFM*	See above for details
	De-familization of ownership	*DFO*	See above for details
Mediating variable	Corporate governance level	*CGL*	See above for details
Moderating Variables	Digital transformation	*Digi*	See above for details
	Market concentration	*Cr4*	See above for details
Control Variables	Company size	*Size*	The logarithm of the enterprise’s total assets.
	Years of listing	*Age*	The current year minus the year of listing
	Debt-to-equity ratio	*Lev*	Total liabilities divided by total assets
	Total asset turnover ratio	*Atr*	Operating Income divided by Average Total Assets
	Current asset turnover ratio	*Cat*	Operating Income divided by Average Current Assets
	Board size	*Board*	Number of Board Members
	Dual leadership roles	*Duarl*	Whether the Chairman and General Manager of the company are the same person. Yes is 1; otherwise, it is 0.
	Executive compensation	*Ec*	The logarithm of the Executive Compensation
	Executive overseas background	*Eob*	Executives in the company have overseas experience (including working or studying abroad) = 1; otherwise = 0.
	Technological Level	*Tech*	Transaction Volume in the Technology Market divided by GDP

### 4.3. Model specification

For testing hypotheses H1a and H1b, we construct the following model:

$${GI}_{it}=\alpha_{0}+\alpha_{1}{DFM}_{it}+\alpha_{2}{DFO}_{it}+\alpha_{4}{Controls}_{it}+{Firm}_{i}+{Year}_{t}+\varepsilon_{it}$$ (1)

*GI*_*it*_ represents the green innovation, *DFM*_*it*_ denotes the level of de-familization in managerial control, and *DFO*_*it*_ signifies the extent of de-familization of ownership. *Controls*_*it*_ denotes the various control variables, α_0_ represents the constant term, *α1*, *α2*, *α3* are coefficients, *Firm*_*i*_ represents individual fixed effects, *Year*_*t*_ represents time fixed effects, and *ε*_*it*_ is the random error term.

To test hypotheses H2a and H2b, we construct the following model based on Model (1):

$${CGL}_{it}=\beta_{0}+\beta_{1}{DFM}_{it}+\beta_{2}{DFO}_{it}+\beta_{4}{Controls}_{it}+{Firm}_{i}+{Year}_{t}+\varepsilon_{it}$$ (2)

$${GI}_{it}=\gamma_{0}+\gamma_{2}{DFM}_{it}+\gamma_{3}{DFO}_{it}+\gamma_{4}{CGL}_{it}+\gamma_{5}{Controls}_{it}+{Firm}_{i}+{Year}_{t}+\varepsilon_{it}$$ (3)

*CGL*_*it*_ represents the corporate governance level of enterprise i in year t, with the meanings of other variables the same as in Model (1).

To test hypotheses H3a and H3b, interaction terms of digital transformation with de-familization (DFM*Digi and DFO*Digi), and interaction terms of market concentration with de-familization (DFM*Cr4 and DFO*Cr4) are included in Model (1).

The definitions of other variables remain consistent with those in Model (1). The adjusted moderating effect model is as follows:

$${GI}_{it}=\delta_{0}+\delta_{1}{DFM}_{it}+\delta_{2}{DFO}_{it}+\delta_{3}{Digi}_{it}+\delta_{4}{DFM}_{it}*{Digi}_{it}+\delta_{5}{DFO}_{it}*{Digi}_{it}+\delta_{6}{Controls}_{it}+{Firm}_{i}+{Year}_{t}+\varepsilon_{it}$$ (4)

$${GI}_{it}=\theta_{0}+\theta_{1}{DFM}_{it}+\theta_{2}{DFO}_{it}+\theta_{3}{Cr4}_{it}+\theta_{4}{DFM}_{it}*{Cr4}_{it}+\theta_{5}{DFO}_{it}*{Cr4}_{it}+\theta_{6}{Controls}_{it}+{Firm}_{i}+{Year}_{t}+\varepsilon_{it}$$ (5)

## 5. Empirical analysis results

### 5.1. Descriptive statistics

Table 2 presents the descriptive statistics for each research variable. The mean of *GI* (0.365) exceeds than the median (0), suggesting that the green innovation of most enterprises falls below the average level. The minimum value of *GI* is 0, and the maximum value is 3.526, indicating significant variation in the green innovation levels among the sampled enterprises. The mean of *DFM* is 0.796, with a median of 0.8, indicating that a large proportion of non-family executives exist in the sample of family firms, demonstrating a certain degree of de-familization. The minimum value of *DFM* is 0.25, and the maximum value is 1, indicating variations in the degree of managerial de-familization among different enterprises, with some companies having all family members completely withdrawing from executive roles. The mean of *DFO* is 0.643, with a median of 0.651, suggesting a substantial proportion of non-family ownership in the sample. The minimum value of *DFO* is 0.055, and the maximum value is 1, indicating significant differences in ownership de-familization among different enterprises. It also implies instances where family shares are completely diluted, aligning with situations where there is a change in the actual controlling shareholders within the sample. Additionally, there are no abnormal conditions observed in other control variables.

**Table 2:** Descriptive statistics.

Variable	N	SD	Mean	p50	Min	Max
GI	786	0.726	0.365	0	0	3.526
DFM	786	0.148	0.796	0.800	0.250	1
DFO	786	0.159	0.643	0.651	0.055	1
Size	786	0.764	21.314	21.269	19.644	24.442
Age	786	3.858	6.683	6	1	25
Lev	786	0.153	0.306	0.288	0.044	0.801
Atr	786	0.208	0.469	0.427	0.079	1.759
Cat	786	0.414	0.851	0.762	0.100	3.012
Board	786	1.355	7.850	8	4	12
Duarl	786	0.496	0.433	0	0	1
Ec	786	0.620	14.857	14.807	12.805	17.253
Eob	786	0.430	0.244	0	0	1
Tech	786	4.580	3.312	1.850	0.067	17.495

### 5.2. Baseline regression results

Table 3 presents the regression results of Model (1). In **Column (1)**, the regression coefficient of *DFM* is -0.6502, significant at the 5% confidence level. This indicates that as the proportion of non-family executives increases, the green innovation level of family firms decreases, implying a negative impact of *DFM* on green innovation. H1a is supported. In **Column (2)**, the regression coefficient of *DFO* is -0.9598, significant at the 1% confidence level. This suggests that a higher proportion of non-family ownership is associated with a lower level of green innovation. Thus, a negative relationship exists between *DFO* and green innovation. H1b is supported. In **Column (3)**, the regression coefficients for *DFM* and *DFO* are -0.5578 and -0.8732, significant at the 5% and 1% confidence levels. This further validates the negative impact of de-familization on green innovation, validating H1a and H1b.

**Table 3:** Regression results.

Variable	(1)	(2)	(3)
	GI	GI	GI
DFM	-0.650**		-0.558**
	(-2.468)		(-2.011)
DFO		-0.960***	-0.873***
		(-3.233)	(-2.712)
Size	-0.028	-0.014	-0.017
	(-0.300)	(-0.151)	(-0.189)
Age	0.021	0.034	0.033
	(1.015)	(1.652)	(1.593)
Lev	-0.262	-0.186	-0.211
	(-0.925)	(-0.697)	(-0.795)
Atr	0.525*	0.562*	0.578*
	(1.715)	(1.815)	(1.906)
Cat	-0.358***	-0.365***	-0.381***
	(-2.916)	(-2.961)	(-3.078)
Board	0.023	0.026	0.028
	(0.865)	(0.973)	(1.075)
Duarl	-0.035	-0.015	-0.041
	(-0.413)	(-0.176)	(-0.486)
Ec	0.049	-0.001	0.039
	(0.639)	(-0.014)	(0.528)
Eob	-0.084	-0.070	-0.081
	(-1.111)	(-0.849)	(-1.016)
Tech	-0.066**	-0.063*	-0.067**
	(-2.115)	(-1.945)	(-2.119)
Firm	Yes	Yes	Yes
Year	Yes	Yes	Yes
_cons	0.737	1.126	1.018
	(0.388)	(0.599)	(0.542)
N	786	786	786
adj. R^2^	0.027	0.030	0.034

Note: This table implies the regression results. Column (1) presents the regression results for DFM and GI. Column (2) presents the regression results for DFO and GI. Column (2) presents the joint regression results of DFM and DFO on GI. The t-statistic is shown in parentheses.

* p < 0.10,

** p < 0.05, and

*** p < 0.01.

### 5.3. Endogeneity issues

We utilize instrumental variable methods(IV) to address potential endogeneity issues between *DFM*, *DFO*, and *GI*. Effective instrumental variables should satisfy the following basic conditions: (1) Relevance, meaning that the IV should be related to both *DFM* and *DFO*. (2) Exogeneity, meaning that the IV should be unrelated to corporate green innovation. Following the approach of Fang et al. (2021), we adopt the average values of *DFM* and *DFO* for other companies within each province and industry (*DFM_IV*, *DFO_IV*) as IV.

Table 4, **Columns (1) and (2)**, display the results of the first-stage regression for the IV test. The results indicate that the P-values for *DFM_IV* and *DFO_IV* are both significant, with a Kleibergen-Paap rk LM statistic P-value of 0.0000, suggesting no identification issues. Furthermore, at the 10% significance level, the values are greater than the critical value (19.93) for the weak IV identification F-test proposed by Stock-Yogo, indicating no weak IV problems. In the results of **Column (3)**, *DFM* and *DFO* are both significantly negative at the 5% and 1% levels, confirming the robustness of Model (1).

**Table 4:** IV test results.

Variable	(1)	(2)	(3)
	DFM	DFO	GI
DFM_IV	-5.797***	0.079	
	(-32.44)	(1.28)	
DFO_IV	0.071	-5.366***	
	(0.77)	(-35.11)	
DFM			-0.590**
			(0.288)
DFO			-1.164***
			(0.307)
Control	Yes	Yes	Yes
Firm	Yes	Yes	Yes
Year	Yes	Yes	Yes
Kleibergen-Paap rk LM statistic	53.542		
Kleibergen-Paap rk Wald F statistic	634.135		
N	786	786	786

Note: This table shows the regression results for the IV test. The t-statistic is shown in parentheses.

* p < 0.10,

** p < 0.05, and

*** p < 0.01.

### 5.4. Robustness tests

#### 5.4.1. Fixed effects

Considering the characteristics of the industry and province where the company is located, such as economic development, legal protection, degree of marketization, and governance decisions of other companies in the same industry, which may all impact the corporate governance structure of local companies

Considering the characteristics of the industry and province where the company is located, such as economic development, legal protection, degree of marketization, and governance decisions of other companies in the same industry [60], we introduce interaction terms of industry and year (Industry*Year), province and year (Province*Year), industry and province (Industry*Province) into Model (1) and re-conducted regression analysis. The results are presented in Table 5.

**Table 5:** Fixed effects test results.

Variable	(1)	(2)	(3)
	GI	GI	GI
DFM	-0.681**	-0.644*	-0.584**
	(-2.483)	(-1.912)	(-2.004)
DFO	-1.068***	-0.977***	-0.988***
	(-3.123)	(-2.804)	(-2.931)
Control	Yes	Yes	Yes
Firm	Yes	Yes	Yes
Industry*Year FE	Yes		
Province*Year FE		Yes	
Industry*Province FE			Yes
_cons	-70.106*	-87.298**	1.347
	(-1.809)	(-2.173)	(0.746)
N	786	786	786
adj. R^2^	0.039	0.003	0.020

Note: This table shows the regression results for fixed effects tests. The t-statistic is shown in parentheses.

* p < 0.10,

** p < 0.05, and

*** p < 0.01.

**Columns (1)-(3)** show that after incorporating interaction term fixed effects into the model, the regression coefficients for *DFM* and *DFO* remain both significantly negative. This result remains consistent with the baseline regression results.

#### 5.4.2. Alternative measures of variables

In this section, we change the measurement method of the dependent variable, green innovation, using the ratio of green innovation output to green innovation input, denoted as *GI1*. Due to the unavailability of data on green innovation investment for listed companies, this paper approximates it by using annual R&D expenditures. The green innovation output is measured using the natural logarithm of the total number of green invention patents applied for by listed companies plus 1. Table 6, **Column (1)**, shows that even after changing the method of measuring green innovation, the inhibitory effect of de-familization on green innovation persists.

**Table 6:** Other robustness test results.

Variable	(1)	(2)	(3)
	GI1	GI	GI
DFM	-0.032**	-1.308**	-0.552*
	(-2.041)	(-2.213)	(-1.965)
DFO	-0.049***	-1.228***	-0.892**
	(-2.628)	(-3.435)	(-2.527)
Control	Yes	Yes	Yes
Firm	Yes	Yes	Yes
Year	Yes	Yes	Yes
_cons	0.064	2.541	0.956
	(0.608)	(0.604)	(0.507)
N	786	393	786
adj. R2	0.033	0.114	0.034

Note: This table shows the regression results for other robustness tests. The t-statistic is shown in parentheses.

* p < 0.10,

** p < 0.05, and

*** p < 0.01.

#### 5.4.3. Adjust the sample range

The Ministry of Industry and Information Technology began publishing SRDI "Little Giants" in 2019. Therefore, the sample range was adjusted to the years 2019–2021, and Model (1) was re-estimated through regression analysis. The regression results in **Column (2)** of Table 6 generally align with those of the baseline regression, indicating the robustness of the findings.

#### 5.4.4. Winsorizing treatment

To mitigate the potential influence of outliers in the dataset, a 1% winsorizing treatment was applied to all continuous variables. The regression results are presented in Table 6, **Column (3)**.

## 6. Mediating effect

Table 7 **Column (1)**, continues to show the significance of Model (1). In **Column (2)**, both *DFM* and *DFO* display a statistically significant positive relationship with *CGL* at the 10% level. Moreover, **Column (3)** indicates a statistically significant negative relationship between *DFM* and *DFO* with *GI* at the 5% and 1% levels, respectively, with coefficients of greater magnitude compared to those in **Column (1)**. Furthermore, a statistically significant positive relationship between *CGL* and *GI* is observed at the 10% level, which implies a mediating role of corporate governance in the relationship between de-familization and green innovation, alongside an observed masking effect. Consequently, Hypotheses H2a and H2b find support.

**Table 7:** Mediating effect.

Variable	(1)	(2)	(3)
	GI	CGL	GI
DFM	-0.545*	0.454*	-0.629**
	(-1.958)	(1.759)	(-2.253)
DFO	-0.892***	0.506*	-0.986***
	(-2.754)	(1.881)	(-3.100)
CGL			0.185*
			(1.814)
Control	Yes	Yes	Yes
Firm	Yes	Yes	Yes
Year	Yes	Yes	Yes
_cons	-0.183	-3.752***	0.511
	(-0.100)	(-2.669)	(0.300)
N	768	768	768
adj. R2	0.035	0.393	0.039

Note: This table shows the regression results for mediating effect. The CGL data for three enterprises in the sample are missing, the number of observations for this test is 768. The t-statistic is shown in parentheses

* p < 0.10,

** p < 0.05, and

*** p < 0.01.

## 7. Moderating effects

After introducing the interaction terms DFM*Digi and DFO*Digi, Table 8, **Column (1)**, reveals that the coefficients for DFM*Digi and DFO*Digi are significant at the 10% and 5% confidence levels, respectively. This finding substantiates the hypothesis that digital transformation mitigates the adverse impact of de-familization on green innovation within SRDI family firms.

**Table 8:** Moderation effects.

Variable	(1)	(2)	(3)
	GI	GI	GI
DFM	-0.685**	-0.565**	-0.680**
	(-2.509)	(-2.056)	(-2.504)
DFO	-0.857***	-0.963***	-0.941***
	(-2.970)	(-3.089)	(-3.322)
Digi	0.103***		0.097***
	(3.206)		(3.039)
DFM*Digi	0.184*		0.177*
	(1.774)		(1.661)
DFO*Digi	0.290**		0.276**
	(2.095)		(2.016)
Cr4		-2.351	-0.787
		(-1.272)	(-0.407)
DFM*Cr4		-5.997**	-5.881**
		(-2.488)	(-2.491)
DFO*Cr4		-25.139***	-24.1945***
Control	Yes	Yes	Yes
Firm	Yes	Yes	Yes
Year	Yes	Yes	Yes
_cons	0.394	0.753	-0.013
	(0.209)	(0.397)	(-0.006)
N	786	786	786
adj. R2	0.035	0.037	0.025

Note: This table shows the regression results for moderation effect. The t-statistic is shown in parentheses.

* p < 0.10,

** p < 0.05, and

*** p < 0.01.

Upon the inclusion of the interaction terms DFM*Cr4 and DFO*Cr4, Table 8 **Column (2)**, demonstrates that the coefficients for DFM*Cr4 and DFO*Cr4 are negative and significant at the 5% and 1% confidence levels, respectively. This result corroborates the proposition that market concentration intensifies the negative association between de-familization and green innovation. Table 8, **Column (3)**, confirms that the empirical findings persist in significance upon the incorporation of all interaction terms, thereby validating Hypotheses H3a and H3b.

## 8. Further analysis

### 8.1. Heterogeneity analysis based on whether selected in the list of "Little Giants"

The cultivation path for SMEs in China follows a trajectory of "innovative SMEs—SRDI SMEs-SRDI Little Giants." Addressing different stages of development along this path, the Chinese government has implemented a series of differentiated policies and incentives in innovation subsidies, tax benefits, financial services, intellectual property, and other areas. Before being selected in the list of "Little Giants", companies were more urgently required to enhance their independent R&D capabilities in their specialized fields, following the developmental path of "innovative SMEs-SRDI" SMEs-Little Giants". As non-family managers and shareholders within the company may have a stronger focus on technological innovation due to their relatively lower emphasis on SEW, the drive for green innovation may be lower. Therefore, the paper anticipates that the negative impact of de-familization governance on green innovation is more significant for companies that have not been selected in the list of "Little Giants" compared to those that have been included. We divide the entire sample into two groups: the selected group and the non-selected group.

Comparing Table 9, **Columns (2)** indicates that, for the sample group of enterprises not selected, both DFM and DFO exhibit statistically significant negative effects. This suggests that the negative impact of de-familization on green innovation is particularly evident in companies not included in the list.

**Table 9:** Heterogeneity analysis results.

Variable	(1)	(2)	(3)	(4)	(5)	(6)	(7)	(8)	(9)	(10)
	Selected	Non-selected	Low-subsidy	High-subsidy	Central	West	North East	East	Key	Non-Key
	GI	GI	GI	GI	GI	GI	GI	GI	GI	GI
DFM	-1.513	-0.550*	-0.358	-1.059*	-2.088	-1.377	0.285	-0.703**	1.446**	-0.725**
	(-1.382)	(-1.769)	(-1.464)	(-1.885)	(-1.575)	(-0.716)	(1.213)	(-2.004)	(2.374)	(-2.254)
DFO	-0.725	-1.074***	-0.758**	-1.422**	-0.832	-0.621	0.032	-0.901***	-0.364	-0.987***
	(-0.541)	(-2.860)	(-2.129)	(-2.291)	(-0.796)	(-0.446)	(0.052)	(-2.670)	(-0.521)	(-2.680)
Control	Yes	Yes	Yes	Yes	Yes	Yes	Yes	Yes	Yes	Yes
Firm	Yes	Yes	Yes	Yes	Yes	Yes	Yes	Yes	Yes	Yes
Year	Yes	Yes	Yes	Yes	Yes	Yes	Yes	Yes	Yes	Yes
_cons	-1.959	-0.473	-1.339	7.069***	7.615	-9.754	-1.881	1.425	5.392*	-2.956
	(-0.172)	(-0.202)	(-0.660)	(2.814)	(1.391)	(-1.410)	(-0.556)	(0.646)	(1.805)	(-1.432)
N	142	644	517	269	84	102	30	570	105	681
adj. R2	0.353	0.029	0.026	0.117	-0.022	-0.000	-0.199	0.042	0.049	0.042

Note: This table shows the regression results for heterogeneity analysis. The t-statistic is shown in parentheses.

* p < 0.10,

** p < 0.05, and

*** p < 0.01.

### 8.2. Heterogeneity analysis based on government subsidies

Shleifer, drawing from the theory of government intervention and the concept of the "grabbing hand," argues that companies, upon receiving government support, are often obliged to adhere to governmental directives, and may even reallocate resources under the government’s "dominance" [61]. China has implemented a comprehensive array of financial subsidies and incentive policies to bolster SRDI enterprises in their specialized domains, facilitating significant advancements and breakthroughs in products and technologies. Consequently, non-family members, who are typically oriented towards short-term gains and profit maximization, are more likely to prioritize the fulfillment of subsidy policy objectives in their decision-making. These individuals are predominantly driven by the pursuit of technological progress, market share, and economic gains. This propensity is anticipated to intensify with the magnitude of government subsidies, potentially redirecting the company’s motivation and resources away from green innovation. We posit that the negative impact of de-familization governance on green innovation will be particularly evident in enterprises with higher levels of government subsidies.

The level of government subsidies is assessed by the ratio of total government subsidies to operating revenue. Government subsidies and operating revenue data are extracted from the annual report, with subsidies specifically identified from the detailed disclosures under "other income" and "non-operating income" sections. The sample is stratified into two groups based on the average level of government subsidies, designated as low-subsidy and high-subsidy groups. As indicated in **Column (4)** of Table 9, there is a significant negative correlation between the regression coefficients of DFM and DFO and enterprises with substantial government subsidies.

### 8.3. Heterogeneity analysis based on region

Based on the differences in China’s economic and social development, we categorize the sample companies into four groups according to their geographical locations: East, Central, West, and Northeast. Initially, the eastern region has experienced earlier reforms and possesses a strategic geographical position, marked by greater advancements in economic development, technological innovation, resource allocation, intellectual property protection, and the establishment of legal frameworks. Consequently, the eastern region offers a more conducive environment for the innovation and development of core business technologies in SRDI enterprises. Subsequently, the eastern region was an early adopter in developing emerging industries and SRDI enterprises. This has led to a higher concentration of SRDI enterprises in the region (Table 9), **Columns (5)-(8)** indicate that there are 570 SRDI enterprises in the eastern region, representing 72.52% of the sample). These enterprises encounter intense competition, particularly in securing market share and acquiring resources. Good conditions and competition push managers and owners to seek technological advances and product improvements. However, non-family members might not value SEW as highly and could allocate resources unwisely, threatening the human and financial capital needed for green innovation. We predict that de-familization’s negative effect on green innovation is stronger in eastern SRDI firms than in those in other areas.

Table 9, **Column (8)** demonstrates that the regression coefficients for DFM and DFO are both statistically significant and negative at the 5% level. These findings substantiate the hypothesis that the negative impact of de-familization on green innovation is more pronounced in SRDI enterprises located in the eastern region.

### 8.4. Heterogeneity analysis based on whether classified as key polluting enterprises

Based on the annual report disclosures regarding the company’s status as a "key pollutant discharge unit" as designated by the Ministry of Environmental Protection, this study categorizes SRDI enterprises into two distinct groups, with the regression outcomes presented in Table 9. **Column (10)** demonstrates that, in contrast to SRDI enterprises listed as key pollutant discharge units, the negative influence of de-familization on green innovation is particularly pronounced in those not included in the list.

Table 9, **Column (9)** indicates that for companies listed as key pollutant discharge units, the DFM coefficient becomes positive and statistically significant, whereas the DFO effect remains insignificant. This implies that these companies may exhibit a more proactive stance on environmental protection, attributed to strong external government supervision and elevated environmental risks. Non-family executives are more inclined to integrate environmental protection measures into their management decisions, thereby contributing positively to the potential for green innovation. Table 9, **Column (10)** reveals that both coefficients are statistically significant and negative. This suggests that non-family executives and shareholders in these companies continue to prioritize product technology innovation, immediate operating profits, and their own interests, with their focus on environmental protection appearing to be lacking. Consequently, the negative impact of de-familization on green innovation is particularly pronounced in these companies, hindering the enhancement of green innovation levels.

## 9. Research conclusions and implications

### 9.1. Research conclusions

This study examines the impact of de-familization on green innovation among A-share listed SRDI family firms from 2016 to 2021, through the lenses of management rights and ownership. The research reveals that both DFM and DFO significantly inhibit green innovation. Corporate governance acts as a mediator in the relationship between de-familization and green innovation, with this effect manifesting as a masking effect. The moderating influence of digital transformation tends to mitigate the negative impact of de-familization on green innovation, whereas market concentration is found to exacerbate this impact. Upon further investigation, the study identifies that the negative impact of de-familization on green innovation is particularly pronounced in companies not yet recognized in the "Little Giants" list, those receiving higher government subsidies, located in the eastern region, or not classified as key pollution control units.

### 9.2. Policy implications

Green innovation stands as a vital component that fosters a "win-win" outcome, enhancing both enterprise competitiveness and environmental protection [62]. Chinese enterprises, particularly those experiencing de-familization within the SRDI category, must proactively engage with the opportunities and challenges presented by green innovation. Accordingly, companies should strategically develop de-familization plans, curtail the myopic behavior of non-family members, enhance their SEW and long-term perspectives, and limit excessive intervention by non-family members in green innovation.

Corporate governance can partially alleviate the negative impact of de-familization on green innovation, yet the situation remains incompletely remedied. Thus, companies should equilibrate the interplay between modern corporate governance and family governance within their management processes, augment supervision and incentives for non-family executives and investors, and bolster their willingness for green innovation.

Advancing green innovation could redirect resources from specialized tech research and impact personal interests. To alleviate concerns of non-family members regarding green innovation, a "dual-track" strategy ought to be implemented. At the internal strategic level, firmly seizing the opportunities of the digital economy is crucial. Digital transformation, when implemented, can improve corporate performance and decrease various costs, thereby bolstering corporate financial resources. Government policies should amplify anti-monopoly efforts to curtail the disorderly expansion and excessive concentration of market structure by dominant entities. This approach will foster the sustainable development of SRDI within a competitive and equitable market landscape.

SRDI family firms should adopt tailored governance strategies to fit their specific needs. Firms not on the ’Little Giants’ list should focus on steady growth, leverage policy benefits, and prioritize green development to join the list. Those with substantial government subsidies should manage these funds wisely to maintain green innovation flexibility. Eastern region companies should leverage their economic and legal advantages to engage in specialized fields and balance R&D efforts in green technologies. For firms not labeled as major polluters, it’s crucial to encourage non-family members to embrace environmental responsibilities in management. Strategic resource allocation in core R&D and green innovation will drive technological advances and eco-friendly practices during the de-familization process.

## Supporting information

S1 Data (DTA)
